# Can We Apply Snyder’s Arthroscopic Classification to Ultrasound for Evaluating Rotator Cuff Tears? A Comparative Study with MR Arthrography

**DOI:** 10.3390/diagnostics13030483

**Published:** 2023-01-28

**Authors:** Marco Porta, Salvatore La Marca, Nicola Carapella, Alessandra Surace, Cristiana Fanciullo, Roberto Simonini, Sandro Sironi, Domenico Albano, Carmelo Messina, Luca Maria Sconfienza, Alberto Aliprandi

**Affiliations:** 1 Department of Radiology, Istituti Clinici Zucchi, 20052 Monza, Italy; 2 Department of Radiology, University of Brescia, Piazzale Spedali Civili 1, 25123 Brescia, Italy; 3 Department of Radiology, ASST Papa Giovanni XXIII Hospital, 24127 Bergamo, Italy; 4 School of Medicine, University Milano Bicocca, 20126 Milano, Italy; 5 IRCCS Istituto Ortopedico Galeazzi, 20161 Milano, Italy; 6 Dipartimento di Scienze Biomediche per la Salute, Università degli Studi di Milano, 20133 Milano, Italy

**Keywords:** Snyder’s classification, rotator cuff tears, shoulder, magnetic resonance arthrography, ultrasound

## Abstract

We aimed to demonstrate the applicability of Snyder’s arthroscopic classification of rotator cuff tears (RCT) in shoulder ultrasound (US) and to compare it with MR arthrography (MRA). Forty-six patients (34 males; mean age:34 ± 14 years) underwent shoulder US and MRA. Two radiologists (R1 = 25 years of experience; R2 = 2 years of experience) assigned A1–4, B1–4, or C1–4 values depending on the extent of RCT in both US and MRA. Inter-reader intra-modality and intra-reader inter-modality agreement were calculated using Cohen’s kappa coefficient. US sensitivity and specificity of both readers were calculated using MRA as the gold standard. Patients were divided into intact cuff vs. tears, mild (A1/B1) vs. moderate (A2–3/B2–3) tears, mild-moderate (A2/B2) vs. high-moderate (A3/B3) cuff tears, moderate (A2–3/B2–3) vs. advanced (A4/B4) and full-thickness (C) tears. The highest agreement values in inter-reader US evaluation were observed for mild-moderate vs. high-moderate RCT (K = 0.745), in inter-reader MRA evaluation for mild vs. moderate RCT (K = 0.821), in R1 inter-modality (US-MRA) for mild-moderate vs. high-moderate and moderate vs. advanced/full-thickness RCT (K = 1.000), in R2 inter-modality (US-MRA) for moderate vs. advanced/full-thickness RCT (K = 1.000). US sensitivity ranged from 88.89%(R1)–84.62%(R2) to 100% (both readers), while specificity from 77.78%(R1)–90.00%(R2) to 100% (both readers). Snyder’s classification can be used in US to ensure the correct detection and characterization of RCT.

## 1. Introduction

Imaging is essential in the comprehensive diagnostic work-up of patients with shoulder pain. Its role in decision-making and preoperative assessment is particularly important when dealing with rotator cuff tears (RCT), being helpful to accurately determine the extent of tears (full-thickness or partial-thickness, bursal or articular-side), thus guiding, together with clinical examination, patient management by the surgeon [1]. RCT are disorders affecting the shoulder mostly due to age-related degenerative changes of the cuff and trauma, with clinical picture ranging from absence of symptoms to severe impairment of life quality [2]. Moreover, this condition is extremely common, with about 25% of patients over 60 years presenting with full-thickness RCT and prevalence further increasing with age [3]. Both ultrasound (US) and magnetic resonance (MR) are accurate techniques in identifying shoulder pathologic conditions and RCT [4,5,6,7]. Compared with US, MR offers a more comprehensive evaluation of the bones, joints, cartilage, and deep soft tissues around the shoulder. MR arthrography (MRA) has slightly higher sensitivity and specificity than both imaging modalities for evaluating RCT, being particularly useful for identifying subtle partial-thickness RCT and is essential for a thorough assessment of the glenoid labrum and glenohumeral ligaments as contrast media distend the joint capsule allowing to better outline intra-articular structures [8,9,10,11].

Nevertheless, US is still extremely important in the evaluation of RCT, especially when performed by highly experienced radiologists, as it is fast, widely available, cheap, and free of ionizing radiations. Several classifications have been proposed for RCT, but none have been applied to US other than just dividing partial or full-thickness RCT [12,13]. Several shoulder orthopedic surgeons use Snyder’s arthroscopic classification of RCT, which includes three parameters: the location of the lesion (bursal- or partial-side), the extent of the lesion (partial-thickness or full-thickness) and the number of involved tendons [14]. Snyder’s classification divides RCT into articular-sided, bursal-sided, and full-thickness tears. The high reproducibility of MRA in evaluating RCT using Snyder’s classification as a method for reporting has been demonstrated, so it would be suitable to be adopted for routine reporting of MRA [15]. Our hypothesis is that Snyder’s classification might be reliably applied to US, thus providing an accurate and more standardized evaluation of RCT, which might be used by orthopedic surgeons to better manage patients with a painful shoulder and, possibly, reduce the request for MR examinations. Hence, the purpose of this study is to evaluate the reliability of shoulder US in the detection and classification of RCT using Snyder’s classification with MRA as the reference standard.

## 2. Materials and Methods

### 2.1. Study Population

Our Institutional Review Board approved this retrospective observational study and waived the need for informed consent (Protocol RETRORAD, Ospedale San Raffaele, Milano, Italy). Our database was completely anonymized to delete any connections between data and patients’ identities according to the General Data Protection Regulation for Research Hospitals. We included all consecutive adult patients who performed shoulder US, followed by shoulder MRA, at our Institution for various diagnostic purposes between June 2021 and January 2022. As our routine clinical practice, Snyder’s classification is used for reporting all shoulder US and MRA examinations. We excluded patients who underwent previous surgery, with incomplete or non-diagnostic MRA examinations, and with extravasation of the contrast agent during injection.

### 2.2. US Protocol

The US evaluation of the rotator cuff at our Institution is standardized following the European Society of Musculoskeletal Radiology (ESSR) guidelines [16]. It was performed with a MyLab X9 echo scanner equipped with a 4–15 MHz linear probe (Esaote, Firenze, Italy). The examination was performed with the patient seated on the bed. All rotator cuff tendons were thoroughly assessed in the short and long axis. Particularly, the patient’s arm was placed posteriorly for evaluating the supraspinatus tendon, placing the palmar side of the hand on the superior aspect of the iliac wing with the elbow flexed and directed posteriorly. The supraspinatus tendon was evaluated along its long and short axis. The intra-articular portion of the biceps was used as a landmark to obtain proper transducer orientation to image the supraspinatus. In fact, these tendons run parallel, and the intra-articular portion of the biceps is easy to recognize due to a more clearly defined fibrillar pattern. Then, the probe was shifted upward and posteriorly over the supraspinatus without changing its orientation. The resulting image is a longitudinal axis with the supraspinatus. By translating the probe by 90°, the short axis of the tendon is obtained. RCT were measured on both planes and were divided into articular-side partial-thickness tears, bursal-side partial-thickness tears, and full-thickness tears.

### 2.3. MRA Protocol

A 1.5-Tesla MR system (Avanto, Siemens Healthineers, Erlangen, Germany) was used with a dedicated shoulder array coil. The patients were placed supine with the shoulder in neutral position, the arm placed along the side, and the thumb pointing upwards [7]. Also, sequences in external and internal rotation of the shoulder have been acquired [17]. All patients provided written informed consent before the procedure. MRA was performed immediately after the intra-articular injection of 20 mL of paramagnetic contrast medium (Dotarem 2.5 mmol/L, Gd-DOTA, Dotarem pre-filled syringes; Guerbet, Paris, France), using anterior approach under ultrasound guidance. After injection, patient’s arm was gently internally and externally rotated for better contrast distribution into the joint capsule. The MRA acquisition protocol parameters are summarized in Table 1.

### 2.4. Analysis of US and MRA Images

Two radiologists, one with 2 years’ experience in musculoskeletal radiology (R2), and the other expert with 25 years’ experience (R1), performed the US examination and retrospectively interpreted MRA. Both radiologists reviewed the MRA examinations and were blinded to US results and clinical data. The two operators assigned a Snyder classification value of A1–4, B1–4, or C1–4, depending on the extent of the lesion [14]. Indeed, at our Institution, all examinations reported by the senior expert have a drafted report previously done by the younger fellow. The interobserver correlation and agreement between US and MRA were calculated using MRA in agreement between the readers as a gold standard.

We grouped Snyder’s lesions of the patients in classes (Table 2):

- 0 negative
- A1–3, B1–3 were regarded as partial-thickness RCT and then subdivided into two sub-classes:
  ○ A1 and B1 as mild RCT
  ○ A2–3 and B2–3 as moderate RCT, then subdivided as:
- Mild-moderate A2 and B2
- High-moderate A3 and B3
- A4, B4, and C1–4, as advanced/full-thickness RCT

### 2.5. Statistical Analysis

We used Cohen’s kappa values to evaluate the agreement between the US results of both radiologists, MRA results of both radiologists, and US and MRA results of the same radiologist (0.000–0.200 poor agreement; 0.201–0.400 fair agreement; 0.401–0.600 moderate agreement; 0.601–0.800 good agreement; 0.801–1.000 excellent agreement). Median values and 95% confidence intervals (CIs) were calculated for the predictive values, kappa values for the comparisons between the results of the US and MRA exams were determined, and McNemar tests were used to test the differences between the US findings reported by the two different radiologists. In addition, MRA was considered the gold standard to calculate the predictive values of US (sensitivity and specificity). Differences between US and MRA measurements were tested for significance with paired t tests. Descriptive statistics were calculated for these tests. Alpha was set at 0.05. Statistical testing was performed using SPSS Statistic Software.

## 3. Results

After applying inclusion and exclusion criteria, 46 patients (34 males, 12 females; mean age: 34 ± 14 years, range 18–65) with shoulder pain were included in our analysis.

The highest agreement values in inter-reader US evaluation were observed for mild-moderate vs. high-moderate RCT (K = 0.745, *p* < 0.001), in inter-reader MRA evaluation for mild vs. moderate RCT (K = 0.821, *p* < 0.001), in R1 inter-modality (US-MRA) for mild-moderate vs. high-moderate and moderate vs. advanced/full-thickness RCT (K = 1.000, *p* < 0.001), and in R2 inter-modality (US-MRA) for moderate vs. advanced/full-thickness RCT (K = 1.000, *p* < 0.001). All data regarding inter-reader intra-modality and intra-reader inter-modality agreements are reported in Table 3.

Considering MRA concordance between both radiologists as the gold standard, the highest values of US sensitivity of both readers, as well as the highest specificity of R2, were reached in the distinction between moderate to advanced/full-thickness RCT (100%). The highest specificity was reached by R1 in the differentiation between mild-moderate vs. high-moderate RCT (100%). All data regarding US sensitivity and specificity of both radiologists using MRA as the gold standard are reported in Table 4.

**Figure 1:**
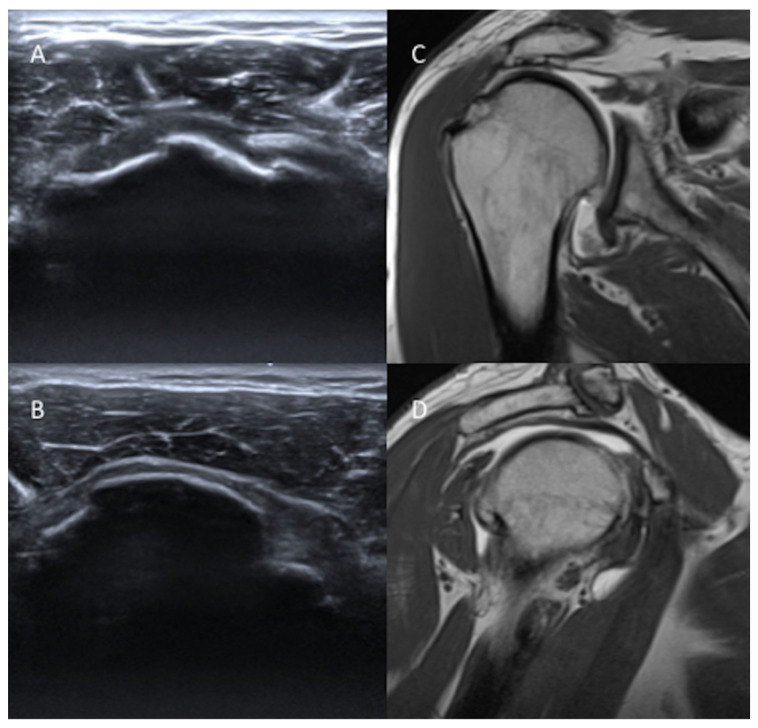
US images on the long axis (**A**) and short axis (**B**), coronal T1-weighted (**C**), and sagittal T1-weighted (**D**) MRA images of a 57-year-old male patient with complete supraspinatus tendon rupture.

Both readers correctly recognized at US the five advanced and full-thickness RCT (Figure 1), but only R1 wrongly interpreted three moderate RCT as advanced/full-thickness RCT. US correctly predicted the width of the full-thickness RCT in four cases, underestimated the width in three, and overestimated it in three. In two of the three cases in which the width was underestimated, the torn cuff was retracted under the acromion.

## 4. Discussion

Our main findings were the moderate to good inter-reader US agreement, the good to excellent inter-reader MRA agreement, and the good to excellent inter-modality agreement of both readers in classifying RCT. Further, very high values of sensitivity and specificity were observed in both readers in describing RCT with US according to Snyder’s classification.

Observer variability is intrinsic to musculoskeletal US, and it can affect diagnostic accuracy. Firstly, proper equipment, including transducer and US parameters selection, is required to optimize results. Secondly, radiologists must adequately evaluate the rotator cuff tendons in a standardized imaging plane, recognize artifacts such as anisotropy, and differentiate them from abnormalities to correctly interpret imaging findings [18]. On MR, variability is primarily related to image interpretation. Minimizing variability depends on proper training, which is common to both US and MR. Several studies have addressed the issue of observer variability with musculoskeletal US. Middleton et al. evaluated the rotator cuff in 61 patients with two observers, both with more than five years of experience, and reported 80% agreement, with perfect agreement concerning full-thickness RCT [19]. Another study compared one observer with six months of experience and another with 15 years of experience in US evaluation of the shoulder and found very good agreement for full-thickness RCT (K = 0.90), moderate agreement for partial-thickness (K = 0.63) and intratendinous (K = 0.57) RCT [20]. Our results are in line with previous literature. However, Martín-Hervás et al. reported 12.5% sensitivity of US for partial-thickness RCT and 57.7% sensitivity for full-thickness RCT, with 67.9% and 100% specificity, respectively [21]. Our sensitivity and specificity were substantially higher, probably due to 20 years of US technology updates which have certainly improved tendon visualization and RCT identification.

Observer variability does exist with MR as well [22]. Robertson et al. reported overall moderate inter-observer agreement and moderate to good intra-observer agreement among four observers. They found good to excellent agreement between readers in the diagnosis of full-thickness RCT but poor agreement in the diagnosis of partial-thickness RCT [23]. Compared to MR, MRA improves diagnostic accuracy and agreement of partial-thickness RCT [8]. Our results are in line with the data reported in the literature. Specifically, excellent inter-observer agreement was obtained by our radiologists on MRA in the differentiation of intact vs. RCT and mild vs. moderate RCT, with substantial superiority of MRA to US, which instead presented moderate to good inter-observer agreement. On the other hand, the differences in inter-observer agreement between US and MRA progressively decreased, moving from mild to severe RCT, with the two modalities showing identical inter-observer agreement in differentiating A2/B2 from A3/B3 (both with K = 0.745) and A2–3/B2–3 from A4/B4/C1-C4 (both with K = 0.734), with US that presented values of sensitivity and specificity ranging from 92% to 100% in both readers. These results are in line with previous radiologic and arthroscopic studies, confirming that a correct identification and perfect classification of location/extent of partial-thickness RCT may be relatively challenging through imaging and arthroscopy as well [24,25]. Unlike previously reported studies, we evaluated partial-thickness RCT separately, confirming that Snyder’s classification, similar to Ellman’s classification [26], is very reliable in distinguishing between mild, mild-moderate, high-moderate or advanced partial-thickness RCT, thus giving the surgeon precise information, as different treatment options can be used, such as debridement in moderate partial-thickness RCT or tendon repair in advanced partial-thickness RCT [27]. Notably, the false positive cases were just related to mild RCT (A1/B1). We can postulate that modern US machines provide such images with higher spatial resolution compared to MRA, making evident subtle tendon irregularities that cannot be clearly depicted by MRA.

A drawback of the Snyder classification is that it does not include interstitial lesions and is limited to the supraspinatus tendon, but our experience in daily clinical practice confirms that radiologists can correctly characterize RCT through this classification, providing clear information that is appreciated by orthopedic surgeons. Some limitations of this study need to be pointed out. First, it is a retrospective observational study, thus we cannot say how the application of Snyder’s classification to US might actually impact treatment decision-making. Second, we did not use arthroscopy results as a reference standard, but MRA is a reliable preoperative imaging modality, as previously reported and also proven by our good to excellent inter-observer agreement. Further, we did not compare US with MRI, which is much more done than MRA, but we have preferred to use MRA as a reference standard given its superiority in the detection of small partial-thickness RCT. Third, the assessment of the reliability of US performed by an orthopedic surgeon could have empowered our analysis, but in our country, shoulder US is rarely performed by orthopedists, who generally use US for a quick and superficial diagnostic evaluation or occasionally for guiding interventional procedures. Then, some CIs are quite wide, specifically the lower limit CI of the inter-rater intra-modality (US) Kappa coefficient was 0.338–0.478, but CI indicates the spread of data and the median value ranges from 0.590 to 0.745, which is the actual data to take into account. Last, the limited number of patients included in our series, although we reached robust statistical results even with a relatively small sample size.

## 5. Conclusions

In conclusion, both MRA and US are well suited for the evaluation of shoulder pain and should be considered complementary imaging modalities for evaluating RCT. In our study, US and MRA have shown similar reliability in detecting and classifying RCT, with better results in full-thickness RCT. This is the first report that demonstrates the applicability of Snyder’s classification on US to be used in clinical practice, even by less experienced radiologists, providing helpful diagnostic findings similar to MRA.

## Figures and Tables

**Table 1:** MRA acquisition protocol.

Sequence	Acquisition Plane	Fat Saturation	Arm Position	Voxel Size (mm)	FoV Read (mm)/Phase (%)	TR/TE (ms)	Slice Thickness (mm)	Distance Factor (%)	Averages/ Concatenations
T1 tse	Axial	No	N	0.6 × 0.6 × 3	200/100	500/11	3	30	1/2
T1 tse	Coronal	No	N	0.6 × 0.6 × 3	190/100	500/11	3	30	1/2
T1 tse	Sagittal	No	N	0.6 × 0.6 × 3	190/100	500/11	3	20	1/2
PD tse	Coronal	Yes	ER	0.3 × 0.3 × 3	190/100	3000/33	3	20	2/1
DP Space	Axial	No	N	0.9 × 0.9 × 0.9	220/100	1000/26	0.9	0	1.4/1
T1 tse fast	Axial	No	IR	0.8 × 0.8 × 3.5	200/90	600/10	3.5	30	1/2
T1 tse fast	Axial	No	ER	0.8 × 0.8 × 3.5	200/90	600/10	3.5	30	1/2

Note- N = neutral, ER = external rotation, IR = internal rotation, FoV = Field of View.

**Table 2:** Adaptation to US of arthroscopic Snyder’s classification of RCT.

Lesion’s Grade	Severity of Partial RCT (A, Articular-Sided or B, Bursal-Sided Lesion)
1	Subtle irregularities of the tendon surface with preserved thickness
2	Major irregularities of the tendon surface with preserved thickness
3	Lesions involve less than 50% of tendon diameter, and lesion extension is less than 3 cm
4	Lesions involve more than 50% of tendon’s diameter with an extension of more than 3 cm, or the lesion involves two tendons
**Lesion’s grade**	**Severity of complete RCT (C)**
1	Small, complete RCT, such as a puncture wound
2	Moderate RCT (usually < 2 cm) that still encompasses only one of the rotator cuff tendons with no retraction of the torn ends
3	Large, complete RCT involving an entire tendon with minimal retraction of the torn edge, usually 3 to 4 cm
4	Massive RCT involving two or more rotator cuff tendons, frequently with associated retraction and scarring of the remaining tendon

Note- RCT = rotator cuff tear.

**Table 3:** Inter-reader intra-modality and intra-reader inter-modality concordance using Cohen’s kappa values (CI 95%). Paired t test was significant for all different classes with *p* <0.001.

Agreement	Intact Rotator Cuff vs. RCT	A1/B1 vs. A2–3/B2–3	A2/B2 vs. A3/B3	A2–3/B2–3 vs. A4/B4/C1–C4
Inter-reader US	0.590 (0.338 to 0.815)	0.694 (0.478 to 0.870)	0.745 (0.454 to 0.945)	0.734 (0.355 to 1.000)
Inter-reader MRA	0.817 (0.627 to 0.957)	0.821 (0.652 to 0.957)	0.745 (0.443 to 0.942)	0.734 (0.364 to 1.000)
US-MRA R1	0.859 (0.685 to 1.000)	0.778 (0.568 to 0.955)	1.000	1.000
US-MRA R2	0.821 (0.618 to 0.956)	0.738 (0.510 to 0.913)	0.862 (0.617 to 1.000)	1.000

Note- RCT = rotator cuff tear; R1 = senior radiologist; R2 = young radiologist.

**Table 4:** US sensitivity and specificity of the two readers (CI 95%) using MRA as the gold standard.

Diagnostic Performance	Intact Rotator cuff vs. RCT	A1/B1 vs. A2–3/B2–3	A2/B2 vs. A3/B3	A2–3/B2–3 vs. A4/B4/C1-C4
Sensitivity R1	96.43% (81.65–99.91%)	88.89% (70.84–97.65%)	92.11% (78.62–98.34%)	100.00% (47.82–100.00%)
Specificity R1	77.78% (52.36–93.59%)	94.74% (73.97–99.87%)	100.00% 63.06–100.00%	92.68% (80.08–98.46%)
Sensitivity R2	89.29% (71.77–97.73%)	84.62% (65.13–95.64%)	97.37% (86.19–99.93%)	100.00% (47.82–100.00%)
Specificity R2	94.44% (72.71–99.86%)	90.00% (68.30–98.77%)	100.00% (63.06–100.00%)	100.00% (91.40–100.00%)

Note- RCT = rotator cuff tear; R1 = senior radiologist; R2 = young radiologist.

## Data Availability

All data are fully available upon reasonable request. The corresponding author should be contacted if someone wants to request the data.
